# Pharmacodynamic Modeling of Acute and Chronic Effects of Methylprednisolone on Hepatic Urea Cycle Genes in Rats*

**Published:** 2008-02-14

**Authors:** Anasuya Hazra, Debra C. DuBois, Richard R. Almon, Grayson H. Snyder, William J. Jusko

**Affiliations:** 1 Department of Pharmaceutical Sciences; 2 Department of Biological Sciences, University at Buffalo, NY 14260; 3 Clinical Pharmacology (Infectious Diseases), Pfizer Inc, New London, CT 06380, U.S.A

**Keywords:** urea cycle, corticosteroids, methylprednisolone, pharmacodynamics, genomics

## Abstract

Corticosteroids (CS) regulate many enzymes at both mRNA and protein levels. This study used microarrays to broadly assess regulation of various genes related to the greater urea cycle and employs pharmacokinetic/pharmacodynamic (PK/PD) modeling to quantitatively analyze and compare the temporal profiles of these genes during acute and chronic exposure to methylprednisolone (MPL). One group of adrenalectomized male Wistar rats received an intravenous bolus dose (50 mg/kg) of MPL, whereas a second group received MPL by a subcutaneous infusion (Alzet osmotic pumps) at a rate of 0.3 mg/kg/hr for seven days. The rats were sacrificed at various time points over 72 hours (acute) or 168 hours (chronic) and livers were harvested. Total RNA was extracted and Affymetrix^®^ gene chips (RG_U34A for acute and RAE 230A for chronic) were used to identify genes regulated by CS. Besides five primary urea cycle enzymes, many other genes related to the urea cycle showed substantial changes in mRNA expression. Some genes that were simply up- or down-regulated after acute MPL showed complex biphasic patterns upon chronic infusion indicating involvement of secondary regulation. For the simplest patterns, indirect response models were used to describe the nuclear steroid-bound receptor mediated increase or decrease in gene transcription (e.g. tyrosine aminotransferase, glucocorticoid receptor). For the biphasic profiles, involvement of a secondary biosignal was assumed (e.g. ornithine decarboxylase, CCAAT/enhancer binding protein) and more complex models were derived. Microarrays were used successfully to explore CS effects on various urea cycle enzyme genes. PD models presented in this report describe testable hypotheses regarding molecular mechanisms and quantitatively characterize the direct or indirect regulation of various genes by CS.

## Introduction

Glucocorticoids (GC) are a class of steroid hormones produced by the adrenal glands that have diverse effects on carbohydrate, lipid, and protein metabolism. While they are important regulators of many developmental processes, immune system and tissue integrity (i.e. bone homeostasis), one of their major physiological roles is the regulation of systemic fuel supplies. This includes gluconeogenesis, the production of glucose from non-carbohydrate sources (primarily glycerol from fat and amino acids from protein). When blood glucose from dietary sources and from breakdown of liver glycogen becomes inadequate, it is the gluconeogenic process that maintains glucose homeostasis. In order to meet this need, GC output occurs in a circadian pattern that mirrors the daily species requirement for increased glucose production. In situations of stress (where energy demands are increased), the adrenal output of GC is increased above normal levels in order to meet the demand.

The effects of GC on the immune system are exploited clinically in the use of synthetic corticosteroids (CS) as immunosuppressive/anti-inflammatory agents. Corticosteroids are widely used to treat a variety of conditions, including organ transplantation, rheumatoid arthritis, lupus, and irritable bowel syndrome. However, such long-term use is associated with serious adverse effects that often reflect a magnification of physiological hormone actions. The development of hypertension, steroid-induced diabetes, atherosclerosis, and muscle weakness can result from chronic CS therapy and to a large extent limits their usefulness as clinical agents. Ascertaining the complex multi-gene, multi-tissue effects of steroid treatment will provide a better understanding of how pharmacologically magnified steroid-induced alterations in gene expression result in complex systemic pathologies.

**Figure 1:**
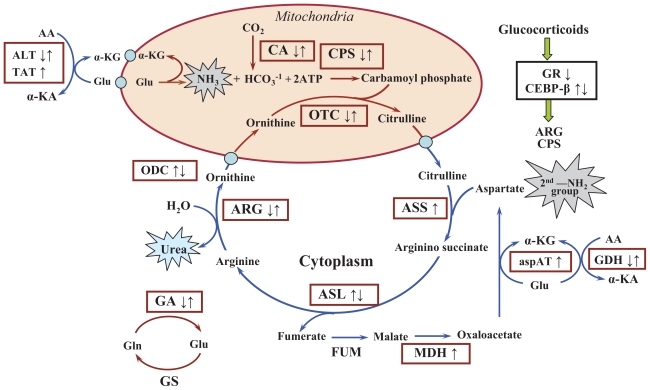
General schematic of greater urea cycle and its regulation by acute and chronic MPL The arrows indicate MPL mediated up- (↑), down- (↓) or biphasic (↑↓: up-followed by down; ↓ ↑: down-followed by up) regulations. **Abbreviations:** AA, Amino acids; ALT, Alanine aminotransferase; ARG, Arginase; ASL, Arginosuccinate lyase; aspAT, Aspartate aminotrans-ferase; ASS, Arginosuccinate synthetase; ATP, Adenosine tri-phosphate; CA, Carbonic anhydrase; CEBP-β, CCAAT/enhancer binding protein; CPS, Carbamoyl phosphate synthetase; FUM, Fumerase; GA, Glutaminase; GDH, Glutamate dehydrogenase; Gln, Glutamine; Glu, Glutamate; GR, Glucocorticoid receptor; GS, Glutamine synthetase; α-KA, α-ketoacids; α-KG, α-ketoglutarate; MDH, Malate dehydrogenase; ODC, Ornithine decarboxylase; OTC, Ornithine transcarbamoylase; TAT, Tyrosine aminotransferase. The enzyme genes in the boxes were analyzed in this study.

Corticosteroid-induced gluconeogenesis and insulin resistance provide the foundation for steroid diabetes. Only two tissues, the liver and kidney, are capable of gluconeogenesis. Gluconeogenesis from amino acid carbon in the kidney requires disposal of the resultant ammonia as ammonium ion. However, in the liver, gluconeogenesis from amino acid carbon requires detoxification of ammonia. This is accomplished in the liver by the urea cycle, which converts ammonia to urea. The general schematic of the urea cycle, adopted from Voet et al. (Voet and Voet, 1990), is shown in Figure 1. Urea synthesis alone requires five enzymes (arginosuccinate lyase, arginosuccinate synthetase, arginase, ornithine transcarbamoylase, and carbamoyl phosphate synthetase). In addition to these five genes, we have defined the “greater urea cycle” and also examined the expression of mRNA for nine additional enzymes that play a role in the disposal of nitrogen in the liver (glutamine synthetase, glutamine dehydrogenase, malate dehydrogenase, carbonic anhydrase, glutaminase, ornithine decarboxylase, tyrosine aminotransferase, aspartate aminotransferase and alanine aminotransferase), and two additional genes involved in transcriptional regulation of those genes; glucocorticoid receptor (GR) and CCAAT/enhancer binding protein (CEBP-β).

Most GC effects are mediated at the molecular level by changes in the expression of specific mRNAs. Gene arrays can provide a method of high throughput data collection that is necessary for constructing comprehensive information on the transcriptional basis of complex systemic polygenic phenomena. When microarrays are used in a rich in vivo time series experiment they yield temporal patterns of changes in gene expression that illustrate the cascade of molecular events in time that mediate broad multi-gene responses. Previously we described the mining and analysis of microarray time series illustrating the responses of liver, skeletal muscle, and kidney taken from the same set of animals to a single bolus dose of methylprednisolone (MPL) (Almon et al. 2005a; Almon et al. 2004; Almon et al. 2005c). These time series included individual chips from multiple control animals as well as multiple animals at each of sixteen times over a 72 hour period following bolus dosing with MPL. Because these experiments were initiated using adrenalectomized animals, the drug in essence acts as a stimulus that perturbs the homeostatic balance of the system, and the deviation of the system and its return to the original state were monitored. Among the genes identified as GC regulated were the 16 genes previously designated as the “greater urea cycle”.

Although very useful, a single time series only views the dynamics of the system in response to the one stimulus. A pharmacological time series differs from most time series studies (for example those assessing developmental changes) in that it can be evaluated using a different dosing regimen. The results from both phases can be used to group genes into clusters with common mechanisms of regulation. If two or more genes have a common mechanism of regulation, then their response profiles should be the same regardless of the dosing regimen. With such data, the challenge then becomes one of constructing rational, quantitative, mechanism-based frameworks that describe the relationships between the elements of the cascades. Kinetic/dynamic modeling provides quantitative, testable, mechanism-based hypotheses concerning the relationship between drug kinetics and elements of cascades that continue long after the drug has dissipated. Such models can accommodate hierarchal cascades where one process generates effectors or mediators for other processes. They can also accommodate convergence of cascades that commonly occur in the control of the expression of genes where binding sites for multiple nuclear factors participate in the regulation of the level of expression of a particular mRNA. This mRNA in turn becomes an endogenous mediator for the expression of proteins that may become effector molecules for other processes. Here, we present the dynamic picture of steroid modulation of expression of the “greater urea cycle” (Fig. 1).

## Materials and Methods

### Experimental

Livers were obtained from animal studies performed in our laboratory (Ramakrishnan et al. 2002; Sun et al. 1998a). In the acute study, male ADX Wistar rats weighing 225–250 g (Harlan Sprague-Dawley Inc., Indianapolis) were subjected to right external jugular vein cannulation under light ether anesthesia one day prior to the study. A single intravenous (IV) bolus dose of 50 mg/kg MPL (Solu-Medrol, Pharmacia-Upjohn Company, MI) was given to 47 animals via the cannula. Rats were sacrificed by aortic exsanguination at 0.25, 0.5, 0.75, 1, 2, 4, 5, 5.5, 6, 7, 8, 12, 18, 30, 48 and 72 hr. The sampling time points were selected based on previous studies of receptor dynamics and enzyme induction in muscle and liver (Ramakrishnan et al. 2002; Sun et al. 1998a; Sun et al. 1998b). Four vehicle-treated rats were designated as controls and were considered as sacrificed at time point zero.

In the chronic-dosing study, 40 male ADX Wistar rats weighing 300–350 g were given 0.3 mg/kg/hr infusions of MPL reconstituted in supplied diluent. The infusions were given using Alzet osmotic pumps (Model 2001, flow rate 1 ul/hr; Alza, Palo Alto, CA). The pump drug solutions were prepared for each rat based on its pre-dose body weight. Pumps were implanted subcutaneously between the shoulder blades on the back. Four rats were sacrificed by aortic exsanguination at 6, 10, 13, 18, 24, 36, 48, 72, 96, and 168 hr. Four control rats were implanted with a saline-filled pump and sacrificed at selected times throughout the 7-day study period.

Blood samples were collected from the abdominal aorta, centrifuged to harvest plasma, and stored at −80 °C until analysis of plasma MPL. Livers were rapidly excised, quickly frozen in liquid nitrogen, and stored at −80 °C.

### Drug assay

MPL plasma concentrations were measured by a previously described normal-phase high performance liquid chromatography (HPLC) method (Haughey and Jusko, 1988). The lower limit of quantification was 10 ng/ml with inter- and intra-day assay variability less than 10%.

### RNA extraction and labeling

Frozen livers were ground into powder using a mortar and pestle chilled with liquid nitrogen. Total RNA extractions were carried out by a Trizol-chloroform based extraction method. About 100 mg of ground liver from each animal was added to prechilled Trizol Reagent (Invitrogen, Carlsbad, CA) at a ratio of tissue: Trizol of 1:10. Extractions were performed according to manufacturer protocols. Extracted RNA was further purified by passage through RNAeasy mini-columns (Qiagen, Valencia, CA). Final extracted RNA samples were resuspended in nuclease-free water. Quantity of total RNA was determined by spectrophotometry and purity was assessed by agarose gel electrophoresis. Extracted total RNA preparations were stored at −80 °C.

### Microarrays

Isolated liver RNA from each individual animal was used to prepare biotinylated cRNA target according to manufacturer protocols. Then biotinylated cRNAs from the acute study were hybridized to 51 (47 treated and 4 control animals) individual Affymetrix GeneChips^®^ Rat Genome U34A (Affymetrix, Santa Clara, CA), which contained 8799 probe sets. The target cRNAs from the chronic study were hybridized to 44 (40 treated and 4 control animals) individual Affymetrix GeneChips^®^ Rat Genome 230A (Affymetrix, Santa Clara, CA), which contained 15967 probe sets. Oligonucleotide microarrays were utilized because of their high reproducibility between separate arrays. The entire data set was submitted to the National Center for Biotechnology Information (NCBI) Gene Expression Omnibus database (GDS253 and GDS972) and is also available online at http://pepr.cnmcresearch.org/ (Almon et al. 2003). Our approach to identifying probes of interest has been described in previously published articles on mining the datasets for muscle, liver, and kidney from the acute MPL treated animals (Almon et al. 2005a; Almon et al. 2004; Almon et al. 2005c). Literature searches of the 1512 probe sets identified as regulated in the acute liver data-set yielded 16 probe sets for 16 genes relevant to ammonia detoxification. Probe sets for these 16 genes were also present on the 230A chip and used to analyze the livers following chronic infusion. These probe sets were statistically examined by ANOVA with a Tukey posthoc test (p < 0.05) using GeneSpring 7.0 software (Silicon Genetics, Redwood City, CA) and were significantly different when compared with the control animals.

Data from both acute and chronic studies were simultaneously modeled. The data for each probe set were transformed so that the values for all probe sets were within the same range. To accomplish this, individual probe set values on each chip were divided by the mean of the four control values for that gene. Thus, the “normalized intensity” for each probe set has a value of 1 at zero time and either decreases, increases, or remains the same when compared to the controls over the time series.

### Pharmacokinetics

The PK of MPL for both the bolus and infusion regimens were described by a two-compartment model with a zero-order input *k*_0_ into the central plasma compartment for the infusion regimen as given below:

(1) $$\begin{array}{l} \frac{{dA}_{p}}{dt}=k_{0}+k_{21}\cdot A_{t}-\left(k_{12}+\frac{CL}{V_{p}}\right)\cdot A_{t}\\ A_{p}(0)=0 \end{array}$$

(2) $$\frac{{dA}_{T}}{dt}=k_{12}\cdot A_{p}-k_{21}\cdot A_{T}\;\;\;A_{T}(0)=0$$

where *A_p_* and *A_t_* are the amounts of drug in the plasma and tissue compartments. The *k*_12_, *k*_21_ and *V_p_* are the distribution rate constants and central volume of distribution; *CL* is clearance. These parameters were fixed based on previous literature values (Ramakrishnan et al. 2002; Sun et al. 1998a). For the i.v. bolus kinetics, the same equations were used with deletion of *k*_0_ and use of *A_p_*(0) = *Dose*. The kinetic parameters were fixed (Table 1) and were used as the driving force for the dynamic modeling.

### Receptor dynamics

**Figure 2:**
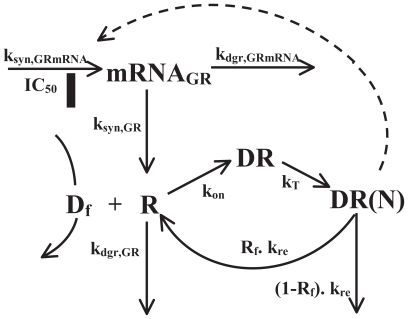
Pharmacodynamic model for glucocorticoid receptor dynamics upon CS administration The symbols are defined in Eq. (3)–(8). The dotted line and the solid rectangle depict inhibition by an indirect mechanism.

Glucocorticoid receptor dynamics in liver following single IV 50 mg/kg MPL dosing was previously described by a mechanistic receptor-gene mediated pharmacodynamic model (Fig. 2). The present model was developed using the assumption that being moderately lipophilic, unbound MPL diffuses through cell membranes and binds with cytosolic free glucocorticoid receptors (GR). Drug-receptor complex (DR) then translocates into the nucleus (nuclear DR: DR(N)) where it dimerizes and binds to a specific glucocorticoid response element (GRE) in the target DNA. The binding of DR(N) and GRE enhances or inhibits the expression of target genes. The CS are known to inhibit the expression of their own receptors (Dong et al. 1988) by homologous down-regulation. After dissociation from DNA, GR receptors are recycled into cytosol, where receptors are either degraded or further activated by MPL (Oakley and Cidlowski, 1993; Sun et al. 1998a). The equations describing the model are:

(3) $$\begin{array}{l} \frac{{dmRNA}_{GR}}{dt}=k_{syn,GRmRNA}\cdot \left(1-\frac{DR(N)}{{IC}_{50}+DR(N)}\right)\\ -k_{dgr,GRmRNA}\cdot {mRNA}_{GR} \end{array}$$

(4) $$\begin{array}{l} \frac{dR}{dt}=k_{syn,GR}\cdot {mRNA}_{GR}+R_{f}\cdot k_{re}\cdot DR(N\\ -k_{on}\cdot C_{MPL,f}\cdot R-k_{dgr,GR}\cdot R \end{array}$$

(5) $$\frac{dR}{dt}=k_{on}\cdot C_{MPL,f}\cdot R-k_{T}\cdot DR$$

(6) $$\frac{dDR(N)}{dt}=k_{T}\cdot DR-k_{re}\cdot DR(N)$$

where symbols represent cytosolic free plasma MPL (*C_MPL,f_*) where the unbound fraction of MPL was fixed to 0.23 (Kong and Jusko, 1991), free glucocorticoid receptor density (*R*), GR mRNA (*mRNA_GR_*), cytosolic GR-drug complex (*DR*), GR-drug complex in nucleus (*DR*(*N*)), zero-order GR mRNA synthesis rate constant (*k_syn,GRmRNA_*), and first-order rate constants for GR mRNA degradation (*k_dgr,GRmRNA_*), GR synthesis (*k_syn,GR_*) and GR degradation (*k_dgr,GR_*). Other rate constants include: second-order association rate constant of GR and drug (*k_on_*), translocation rate constant of *DR* from cytosol to nucleus (*k_T_*), and the overall turnover rate of *DR*(*N*) (*k_re_*). The fraction of GR that is recycled from nucleus to cytosol (*R_f_*) can be further activated by association of drug. Thus (*1*−*R_f_*) is the fraction of GR that is degraded during one cycle of drug-receptor complex translocation. The *IC*_50_ is the concentration of *DR*(*N*) at which the synthesis rate of GR mRNA is reduced to 50% of its baseline level.

Assuming that in the absence of drug the *mRNA_GR_* remains constant, the synthesis rate is:

(7) $$k_{syn,GRmRNA}=k_{dgr,GRmRNA}\cdot {mRNA}_{GR}(0)$$

Also in the absence of drug assuming that *R*_0_ is constant, the translational rate constant *k_syn,GR_* is:

(8) $$k_{syn,GR}=\left(\frac{R_{0}}{{mRNA}_{GR(0)}}\right)\cdot k_{dgr,GR}$$

The values for the baselines of both the GR mRNA and the receptors were fixed and were obtained from Sun et al. (Sun et al. 1998a; Sun et al. 1998b). The baseline values for *DR* and *DR*(*N*) are zero. The detailed analysis of receptor dynamics (parameters given in Table 1) were done elsewhere (Hazra et al. 2007) and were fixed to drive further dynamic modeling.

### Pharmacogenomic modeling

In the present report we measured drug kinetics, GR concentration, GR mRNA concentration, and the normalized concentrations of 16 mRNAs, some of which are transcription factors that in turn become factors controlling the expression of genes. Since mRNAs have natural turnover processes, we have used various inhibitory or stimulatory models (Dayneka et al. 1993) to describe the primary or secondary regulation of these genes by CS.

Most of the metabolic effects of CS have been attributed to its classical model of action where binding of nuclear hormone-bound GR complexes to the regulatory regions of various target genes causes stimulation or inhibition of expression of these genes. Many of the genes related to nitrogen metabolism are known to have GREs in their promoter regions and therefore presumably are directly affected by binding of the nuclear drug-bound receptor to these regions. However, it has also been found that CS causes changes in gene as well as protein expression of transcription factors such as C/EBP-β or HNF-3 (Gotoh et al. 1997; Morris, 1992; Schoneveld et al. 2004) which are also strong regulators of various urea cycle genes. As a result both primary (mediated by GR binding GREs in promoter regions of target genes) as well as the secondary transcriptional regulation are passive mechanisms for GC modulation of mRNA expression.

Both mechanisms were taken into consideration to develop models to describe the dynamics of various genes related to the greater urea cycle after acute and chronic MPL. Many of the genes showing simple up- or down-regulation after acute dosing seemed to have complex patterns after chronic dosing. Our aim was to build models which were able to simultaneously describe the time profiles of these genes after both treatments.

In the absence of drug, all the genes were assumed to be synthesized at a zero-order rate (*k_syn,Enzm_*) and degraded with a first-order rate constant (*k_dgr,Enzm_*) as follows:

(9) $$\frac{dmRNA}{dt}=k_{syn,Enz_{m}}-k_{dgr,Enz_{m}}\cdot mRNA$$

Since the rats were adrenalectomized, the genes under consideration were assumed to have a stationary baseline (*mRNA*_0_) in the absence of the drug (control animals), thereby allowing *k_syn,Enzm_* to be calculated from:

(10) $$k_{syn,Enz_{m}}=k_{dgr,Enz_{m}}\cdot {mRNA}_{0}$$

All mRNA baselines were fixed to 1.0 except for the cases where estimation of this parameter improved the model fittings significantly (assessed by visual inspection and AIC, SC criteria).

### Stimulation of transcription

**Figure 3:**
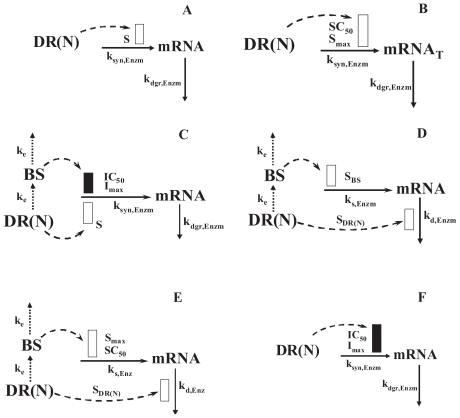
Pharmacogenomic models for CS effects on genes related to urea cycle via various mechanisms Models A to F are defined in detail in the Methods section and with Eq. 9–16. The rectangles indicate stimulation (open bar) and inhibition (solid bar) of the various processes via indirect mechanisms.

This characteristic was assumed to be mediated by the stimulatory effect of *DR*(*N*) on the synthesis rate of mRNA. Two conditions may arise; either the stimulation by *DR*(*N*) may be linear, non-saturable (Fig. 3A) in nature for a given gene or dose of the drug (Eq. 11a) or it could be a saturable function (Fig. 3B) where the effect of the drug is limited by capacity-limited factors such as *S_max_* and *SC*_50_ (Eq. 11b) as given by:

(11a) $$\begin{array}{l} \frac{dmRNA}{dt}=k_{syn,Enz_{m}}(1+S\cdot DR(N))\\ -k_{dgr,Enz_{m}}\cdot mRNA \end{array}$$

(11b) $$\begin{array}{l} \frac{dmRNA}{dt}=k_{syn,Enz_{m}}\left(1+\frac{S_{\text{max}}\cdot DR(N)}{{SC}_{50}+DR(N)}\right)\\ -k_{dgr,Enz_{m}}\cdot mRNA \end{array}$$

where S is a linear stimulation constant by which DR(N) increases the synthesis of the enzyme mRNA, *SC*_50_ is the concentration of *DR*(*N*) responsible for 50% of maximum stimulation, and *S_max_* is the maximum stimulation capacity.

### Biphasic regulation

Two different models were developed to describe biphasic regulation. In the first model as depicted in Figure 3C, *DR*(*N*) linearly stimulates the production of the enzyme mRNA whereas a biosignal (BS) derived from *DR*(*N*) is responsible for secondary inhibition of the production as given by:

(12) $$\frac{dBS}{dt}=k_{e}\left(DR(N)-BS\right)$$

(13) $$\begin{array}{l} \frac{dmRNA}{dt}=k_{syn,Enz_{m}}(1+S\cdot DR(N))\\ \cdot \left(1-\frac{{BS}^{\gamma }}{{IC}_{50}^{\gamma }+{BS}^{\gamma }}\right)\\ -k_{dgr,Enz_{m}}\cdot mRNA \end{array}$$

where *k_e_* is a first-order transduction rate constant accounting for the delayed formation of *BS* from *DR*(*N*), *S* is a linear stimulation constant by which DR(N) stimulates the production rate constant, *IC*_50_ is the concentration of *BS* responsible for 50% inhibition of the mRNA synthesis rate, *γ* is a factor describing amplified effects of *BS* on the production of mRNA. In the absence of the drug, the *BS* value is set to zero.

In the second model, described in Figures 3D and 3E, the *DR*(*N*) increases the degradation of mRNA, whereas the biosignal (Eq. 12) derived from the *DR*(*N*) is responsible for a delayed increased (either linear or non-linear) production of the mRNA as given by:

(14) $$\begin{array}{l} \frac{dmRNA}{dt}=k_{s,Enz}\left(1+S_{BS}\cdot BS\right)\\ -k_{d,Enz}(1+S_{DR(N)}\cdot DR(N))mRNA \end{array}$$

(15) $$\begin{array}{l} \frac{dmRNA}{dt}=k_{s,Enz}\left(1+\frac{S_{max}BS}{{SC}_{50}+BS}\right)\\ -k_{d,Enz}(1+S_{DR(N)}\cdot DR(N))mRNA \end{array}$$

where *S_BS_* is the linear constant, *S_max_* and *SC*_50_ are the capacity-limited stimulatory constants by which *BS* increases the production rate constant, and *S_DR_*_(_*_N_*_)_ is the linear stimulation constant by which *DR*(*N*) regulates the degradation rate constant for mRNA.

### Down-regulation

As depicted in Figure 3F, down-regulation of mRNA was described by:

(16) $$\begin{array}{l} \frac{dmRNA}{dt}=k_{syn,GR}\left(1-\frac{DR(N)}{{IC}_{50}+DR(N)}\right)\\ -k_{dgr,GR}\cdot mRNA \end{array}$$

where *IC*_50_ is the *DR*(*N*) concentration at which mRNA synthesis rate reduces to 50% of its baseline value.

### Scaling Factor

Since two types of chips were used for the acute and chronic studies, a factor (*SF*) was incorporated in the chronic study data (*Y_chronic_*) to account for the possible difference in sensitivity between the probe sets (Yao et al. 2007):

(17) $$Y_{chronic}={\left({mRNA}_{chronic}\right)}^{SF}$$

where *mRNA_chronic_* is the data (from 230A chips) for the gene of interest after chronic infusion of MPL.

Data for each gene from both the acute and chronic studies were fitted simultaneously with ADAPT II software (D’Argenio, 1997) using the Maximum Likelihood method, where the variance model was described as:

(18) $$Var\left(\sigma ,\theta ,t_{i}\right)=\sigma_{1}^{2}\cdot Y{\left(\theta ,t_{i}\right)}^{\sigma^{2}}$$

where *Y* represents the predicted value; *σ*_1_ and *σ*_2_ are the variance parameters which were fitted, and *θ* represents the structural parameters. The goodness of the fit was assessed by model convergence, visual inspection, Akaike Information Criterion (AIC), Schwarz Criterion (SC), estimator criterion value, and examination of residuals.

## Results

### Pharmacokinetics and receptor dynamics

**Figure 4:**
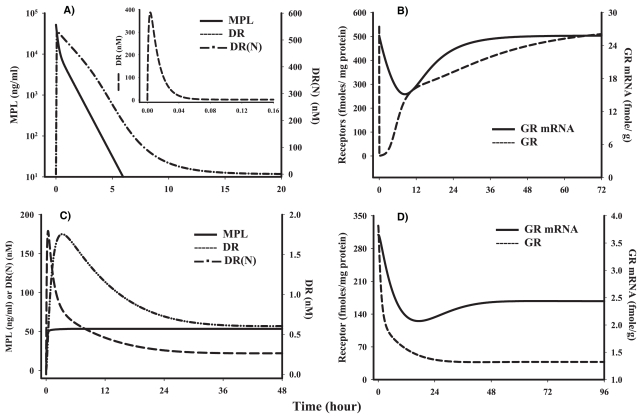
Time profiles of various components in MPL pharmacokinetics and receptor dynamics Lines are simulations with the model given in Figure 2 after 50 mg/kg MPL i.v. injection (4A and B) or 0.3 mg/kg/hr chronic infusion for seven days (4C and D) in ADX rats using parameters listed in Table 1.

The experiments were initiated by MPL dosing. Although the kinetic data for both regimens have been previously published (Ramakrishnan et al. 2002; Sun et al. 1998a), simulated pharmacokinetic profiles for both are provided as they represent the initial driving force for the dynamic models (Fig. 4). Figure 4a shows the simulations of MPL kinetics, DR, and DR(N) following the acute dose of MPL. Although the experiment continued for 72 hours, the figure is truncated at 20 hours because MPL reached its lower limit of quantification by 6 hr and the drug-receptor complexes returned to their baseline prior to this time. Figure 4b shows the simulation of GR and GR mRNA which is also based on previously published data (Sun et al. 1998b). Figure 4c shows the simulations of MPL kinetics, DR, and DR(N) during chronic infusion of MPL. This graph only shows the first 48 hours of the 168 hour infusion period because all three variables reached a steady-state by this time. Figure 4d shows the simulation of GR and GR mRNA during chronic infusion which is also based on previously published data (Ramakrishnan et al. 2002). The steady-state concentration after the chronic dosing (i.v. infusion) was 100-fold less than the bolus dose study. The clearance (3.48 to 5.61 L/hr) difference could be attributed to the saturation of the metabolizing enzymes at the high dose. The *DR*(*N*) is used as the driving force regulating many of the genomic effects of CS.

### Pharmacogenomic modeling

The dynamics of most of the genes showed more complex regulation during chronic infusion of MPL compared to acute bolus dosing. Based on the time profiles from the acute dosing study the genes of the greater urea cycle could be divided into three main classes, up-regulated, biphasic, and down-regulated. These could be further divided into various sub-categories based on the chronic dataset.

#### Class 1

This class is comprised of four genes which were up-regulated in response to both acute and chronic administration of MPL. Model A or B (shown in Fig. 3), where the transcription is assumed to be increased by *DR*(*N*), was used to capture the general trend of the data (Fig. 5). The dynamics of tyrosine aminotransferase (TAT) was described by Model A as discussed in previous reports based on measurements by other methods (Ramakrishnan et al. 2002; Sun et al. 1998a). The degradation rate constant *(k_dgr,Enz_)* for TAT mRNA, 0.503 hr^−1^ closely resembled the reported value of 0.383–0.533 hr^−1^ (Ramakrishnan et al. 2002; Sun et al. 1998a). The other three genes in this class, aspartate aminotransferase (aspAT), argininosuccinate synthetase (ASS), and malate dehydrogenase (MDH), were described more accurately by Model B with capacity-limited stimulation by *DR*(*N*). The apparent tolerance observed for TAT and aspAT (Fig. 5a) was not observed for ASS or MDH (Fig. 5b), which can be explained by much lower values for *SC*_50_ (Table 2) for the latter two genes (4.1 and 14.0 nM) compared to aspAT (107.4 nM). The variation in the degree of stimulation (normalized signal ranging from 2.5–12) of the messages was also reflected by their respective *S_max_* values, ranging from 0.76 to 7.3 (fmol/mg protein)^−1^. The precisions of the estimated parameters were reasonable with the exception of the *SC*_50_ values for ASS and MDH. The scaling factors for all four genes ranged between 1 and 3 and were estimated with good precision.

#### Class 2

**Figure 6:**
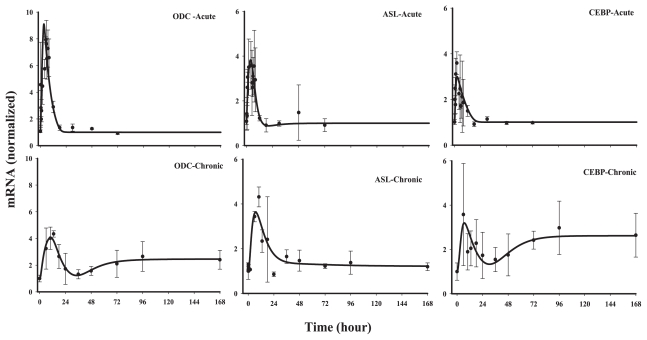
Fittings of *Class 2* genes Solid circles are the mean data with standard deviations. Solid lines are fittings with Model C (Eq. (12)–(13)) for each individual gene after MPL administration. The estimated parameters are listed in Table 3.

The genes in this group are also up-regulated with acute dosing, but show a biphasic pattern with chronic dosing. The biphasic pattern is more apparent for ornithine decarboxylase (ODC) and CCAAT/enhancer binding protein (CEBP-β) than ASL, suggesting possible existence of dual mechanisms in the regulation of these genes. Model C, where the initial increase in the message is assumed to be mediated by *DR*(*N*) followed by a decline driven by a biosignal or a feedback regulator, best described (among other models tested) the general trend of the data for all three genes (Fig. 6) with relatively good precision for the estimated parameters. Since the biphasic trend is not quite apparent for argininosuccinate lyase (ASL), Models A and B were tested for this gene, however, Model C resulted in best fit of the data from visual inspection and model selection criteria such as AIC and SC values. The model-fitted parameters for all three genes are given in Table 3. Since the transduction parameter, *k_e_* for CEBP-β could not be estimated with reasonable precision, it was fixed to an optimum value of 0.1 (upon performing sensitivity analysis).

The degradation rate constant, *k_dgr,Enzm_* for ODC (0.14 hr^−1^) was estimated to be somewhat lower than previously reported (0.3 hr^−1^) by Jin et al. (Jin et al. 2003), however, use of a different as well as more complex model could contribute to this difference. Although the *IC*_50_ values for inhibition of the message by the biosignal were quite similar between these genes (ranging from 65–76.6 nM), the values for the amplification factor, *γ*, were much higher than 1.0 and had significant variation ranging from 3.9 for ASL to 14.7 for ODC. The scaling factors for this group of genes were close to 1.0 with CV less than 20%.

#### Class 3

**Figure 8:**
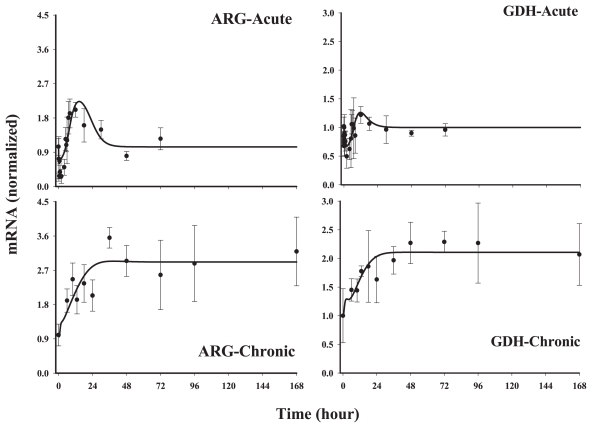
Fittings of *Class 3* genes Solid circles are the mean data with standard deviations. Solid lines are fittings with Model E (Eq. (12)–(15)) for each individual gene after MPL administration. The estimated parameters are listed in Table 4.

This group of five genes showed an early down-regulation following acute MPL and a later and sustained up-regulation. However, during chronic infusion stimulation of the messages was predominant, except for glutamate dehydrogenase (GDH) and ornithine transcarbamoylase (OTC). It is important to note that for chronic infusion, the first sampling was at 6 hr following initiation of infusion. Therefore, it is possible to miss the initial down-regulated phase seen in the acute response profiles. Models D and E, where the nuclear drug-receptor complex (*DR*(*N*)) mediates the initial decline followed by an up-regulation caused by a mediator, *BS*, derived from *DR*(*N*), could adequately capture the biphasic patterns of these genes both after acute and chronic administration. A linear stimulation constant (Model D) was sufficient to describe the biosignal mediated increase in OTC, alanine aminotransferase(ALT) and carbamoyl phosphate synthetase (CPS). However, similar to genes in *Class 1* (ASS and MDH), we observed a lack of tolerance in arginase (ARG) and GDH. Model E with saturable stimulation factors (*S_max_* and *SC*_50_) yielded better fittings compared to Model D for these two genes when evaluated by various fitting criteria. The estimated parameters from model fittings are shown in Table 4. For the three genes which were characterized by Model D, most of the estimated parameters, especially *k_dgr,Enzm_* (0.034–1.0 hr^−1^), *S_DR_*_(_*_N_*_)_ (0.004–0.027 nM^−1^) and *k_e_* (0.03–1.1 hr^−1^) varied between the genes, however, the parameters estimated were usually associated with higher CV%. For the two genes, arginase and GDH (Fig. 8), which could be best described by Model E, the *SC*_50_ parameters could not be estimated with reasonable precision and were fixed to optimum values based on the various fitting criteria. Due to the variability associated with data for GDH, both *k_e_* and *SC*_50_ values could not be estimated with good precision and therefore were fixed based on the estimates for the rest of the parameters. Thus the apparent high values for *k_e_* (10 hr^−1^) and *SC*_50_ (100 nM^−1^) for GDH should be interpreted with caution. One of the primary reasons for the high CV% of the estimated parameters could be attributed to possible over-parameterization of the model with the limited amount of data.

#### Classes 4A and 4B

**Figure 9:**
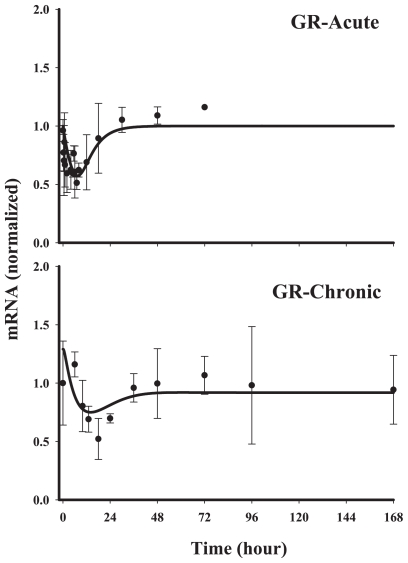
Fitting results of gene array data for glucocorticoid receptor Solid circles are the mean data with standard deviations. Solid lines are fittings with Model F (Eq. (16)) after MPL administration. The estimated parameters are listed in Table 5.

**Figure 10:**
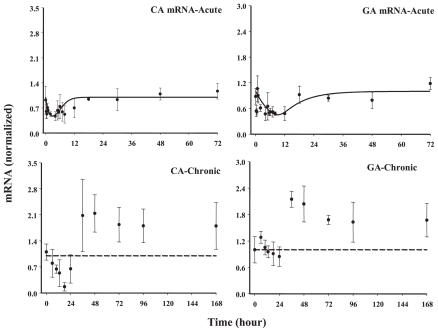
Fittings of *Class 4B* genes Solid circles are the mean data with standard deviations. Solid lines are fittings with Model F (Eq. (14)) for each individual gene after MPL administration. The estimated parameters are listed in Table 5.

Acute dosing of MPL causes down-regulation for this class of genes which can be captured by Model F where *DR*(*N*) inhibits the production rate (*k_syn, Enzm_*) of the mRNA. For glucocorticoid receptor (GR) we could observe (Fig. 9) similar down-regulation upon chronic infusion thus acute and chronic dataset could be fitted simultaneously (Class 4A) using Model F with good fitting results evaluated by various criteria. For glutaminase (GA) and carbonic anhydrase (CA), although an apparent down-regulation was observed after acute administration without any indication of a biphasic pattern, chronic infusion revealed a delayed and sustained up-regulation (Fig. 10) in the gene profiles. This suggests an existence of dual mechanisms after continuous exposure of the drug. No suitable model could explain these differences simultaneously and therefore, only the acute dataset were fitted with Model F. The estimated parameters for these three genes are given in Table 5. The estimated degradation rate constant for GR mRNA was very similar to previously reported values based on data from Quantitative Northern Hybridization measurements (Sun et al. 1998a; Sun et al. 1998b). Most of the parameters could be estimated with reasonable precision with CV% less than 30%.

## Discussion

Corticosteroids, either alone or in conjunction with transcription factors/hormones affect a wide array of genes responsible for regulating various physiological processes. Recently oligonucleotide microarrays were successfully used in our lab to examine these diverse effects of corticosteroids from two sets of animal studies, which allowed us to evaluate changes in an enormous number of genes in various tissues simultaneously after acute (Almon et al. 2004; Almon et al. 2005c; Jin et al. 2003) and chronic MPL. While such evaluations are quite helpful in determining which genes are affected by steroids in rats, another important aspect of such experiments is specific disease/physiological process based evaluation of the data as demonstrated by Almon et al. who examined the effects of MPL on insulin resistance specific genes (Almon et al. 2005b) in rat muscle. In this report, we investigated the dynamics of various genes related to the greater urea cycle, an important biochemical pathway present in mammalian livers for nitrogen disposal. Many of these enzymes are known to be highly regulated by CS in the liver (Christowitz et al. 1981; de Groot et al. 1984; Gautier et al. 1985).

The dynamics of many of these genes following acute dosing were previously reported from our lab (Jin et al. 2003). However simultaneous analyses of both the acute and chronic dataset revealed more complex gene regulations which were not apparent after only single high-dose MPL.

*Class 1* and *Class 4A* were the simplest regulation patterns apparent amongst all the classes. TAT, which facilitates the transfer of an amino group from amino acid breakdown to the urea cycle and is also one of the most highly studied biomarkers, could be classified as one of the *Class 1* genes. Both TAT and glucocorticoid receptor (*Class 4A)* dynamics had extensively been studied in our lab (both at mRNA and protein levels) and five generations of highly mechanistic, quantitative PK/PD models were developed for MPL at various dose levels (Ramakrishnan et al. 2002; Sun et al. 1998a; Sun et al. 1998b). Thus the presence of these two genes in our database not only had importance with respect to their involvement in the urea cycle, but also allowed us to compare the results from the microarrays to previous measurements by quantitative Northern Hybridization. For TAT the correlations (R) between these two techniques were quite high for both acute (0.68) and chronic (0.87) dosing while for GR the correlation was less than 0.5 which could be attributed to the much lower abundance of this message, the presence of greater variability in these data, and the more limited downward range of observed changes.

The other *Class 1* genes were aspAT, with a similar function as TAT, ASS, one of the five urea cycle enzymes and MDH, a citric acid cycle enzyme that catalyzes the conversion of malate into oxaloacetate, thus facilitating recycling of the urea cycle by-product fumerate, which are shown in the urea cycle general schematic in Figure 1. The dynamic profile for aspAT (Fig. 5), known to be regulated by CS (Garlatti et al. 1996; Pave-Preux et al. 1988) resembled TAT, however, incorporation of a capacity-limited stimulation by *DR*(*N*) seemed to provide the best fit for the data.

The action of CS on ASS and MDH are known to be secondary (Husson et al. 2003; Recupero et al. 1986; Ulbright and Snodgrass, 1993). Bourgeois et al. reported dose-dependent increase in ASS mRNA expression in cultured rat hepatocytes which could be completely abolished by actinomycin D indicating involvement of the synthesis of some secondary factors in this regulation (Bourgeois et al. 1997). The MDH has also been reported to be secondarily regulated by CS by potentiating the binding capacity of T3 receptors since CS action on MDH in thyroidectomized animals was minimal (Recupero et al. 1983; Recupero et al. 1986). However, our current *in vivo* datasets precluded addition of such secondary factors and therefore for simplicity Model B was used to capture both these enzyme inductions. For both ASS and MDH, however, the general patterns of the stimulation were quite different than TAT or aspAT. While the acute time profiles for ASS and MDH yielded a somewhat delayed and prolonged stimulation (lower degradation rates, 0.15 and 0.08 hr^−1^ compared to TAT and aspAT, 0.506 and 0.81 hr^−1^), chronic infusion profiles for these two genes did not follow the apparent tolerance phenomenon visible in the chronic *DR*(*N*) profile (Fig. 4). Rather the message seemed to remain up-regulated during the entire period of seven days (Fig. 5) indicating somewhat higher sensitivity to *DR*(*N*) compared to aspAT or TAT. The estimated values of the *SC*_50_ parameters for these two genes (Model B) agreed with this observation, almost 15–20 fold (3.2–3.5 nM) lower than the steady-state *DR*(*N*) after chronic MPL (57 nM) compared to the higher estimated value for aspAT (191.5 nM).

Reports in the literature show CS-mediated direct or indirect increases in all three genes of *Class 2,* i.e. ASL, CEBP-β and ODC (Chowdhury et al. 1996; Hirvonen et al. 1988; Matsuno et al. 1996; Nebes and Morris, 1988). Although our acute dataset predominantly showed up-regulation for these genes, chronic profiles indicated biphasic responses, especially for ODC and CEBP-beta. The initial increase in these messages was followed by a down-regulation up to or close to their baseline followed by a delayed increase. This was similar to the *Class 1* genes suggesting a possible existence of a negative feedback regulator limiting the degree of increase of the message during chronic infusion of MPL. Models with a secondary mediator arising from the message itself regulating the initial increase would probably be most mechanistic; however, model convergence could not be achieved with such a model. ASL, one of the five urea-cycle enzymes known to be secondarily regulated by CS, showed similar profile as TAT or aspAT, however incorporation of the biosignal (Model C) was necessary for satisfactory fitting of the data.

An important transcription factor CEBP-β, contains a GRE in its promoter region and has been postulated to be one of the primary mediators of glucocorticoid effects on urea cycle enzymes in the liver, especially arginase and CPS. This chain of effects, i.e. of stimulation of CEBP-β by CS and in turn delayed stimulation of ARG and CPS by CEBP-β could be nicely captured for the acute dataset (results not shown); however, this model could not be implemented for the chronic dataset where the regulation seemed to be more complex, perhaps due to the involvement of multiple regulatory factors.

*Class* 3 contains the largest number of genes among all the classes (including three of the five main urea cycle enzymes) predominantly showing a biphasic response, usually a rapid down-regulation followed by an up-regulation, which could generally be described by Model D or E. Such a phenomenon is not surprising for CS since they may induce or inhibit the same gene depending on other regulatory proteins/transcription factors that are already present on the DNA control regions (Alberts et al. 2001). Since the up-regulation was somewhat delayed for all the genes, both models assume the increase is mediated by a biosignal.

Although most of the reports in the literature suggest an inhibitory effect of CS on OTC in fetal as well as in adult rats (Gautier et al. 1985; Gautier et al. 1977; Ulbright and Snodgrass, 1993), we observed a subtle and delayed up-regulation in this gene after both acute and chronic MPL. As has been mentioned in the previous section, the coordinated regulation of CS on CPS (the first enzyme of the ornithine cycle, converts ammonia, originating from amino acid degradation, into urea) or arginase (the last enzyme of the cycle) via CEBP-β could not be applied to the chronic data-set and thus these two genes were modeled independently of CEBP-β. Arginase and GDH seemed to show saturability in stimulation by the biosignal similar to a few of *Class 1* genes. Although this phenomenon could be described by Model E, the precision of the estimated parameters was poor perhaps because of over-parameterization of the model and inadequate data thus limiting the capability of the estimation, especially the capacity-limited parameters such as *SC*_50_.

*Class 4B* genes, unlike *Class 3* genes, do not show any delayed stimulation after acute MPL, however chronic dosing predominantly showed delayed up-regulation (glutaminase, involved in interconversion of two amino acids, glutamine and glutamate) or a biphasic response (carbonic anhydrase, responsible to form the bicarbonate ion from carbon dioxide from the Kreb’s cycle for the first step of urea cycle) suggesting differential regulation between acute and chronic dosing. Not much information is available regarding CS regulation of these two genes in the literature; however, initial investigation of the acute dataset showing down-regulation only was surprising to us owing to the fact that CS treatment seemed to increase most of the genes related to the urea cycle either instantaneously or with a delay, which could be clearly observed only with chronic dosing for these two genes.

An important component of our analysis of both acute and chronic dosing of MPL was the necessity of including a scaling factor for the chronic dataset. Since this factor was only included in the chronic dataset (refer to Eq. 17), a value of greater than 1.0 signified greater sensitivity for the chips for the chronic dataset. Two different chips were used for the two treatment groups and data generated from them may not always produce concordant results. In general signal values tend to be higher for the majority of RAE-230A probe sets relative to the corresponding probe sets on RG-U34 suggesting a need for a scaling factor to accommodate this discordance. It was suggested by Affymetrix that the newer 230A chip is most likely to outperform the RG-U34 probe set when both magnitude of signal and responsiveness to a biologically diverse tissue panel are evaluated. The 230A chips were superior in 51% of the probe sets, 5.7% of the probes seemed to have higher signals in RG-U34 chips, and less than 50% of the probes had equivalent signals.

Consistent with the physiological role of glucocorticoids in stimulating gluconeogenesis/urea cycle activity, most urea cycle genes exhibited a general enhanced expression following CS treatment. However, this rich time series data set illustrates that examination of changes in gene expression at a single time following drug administration can be misleading as to magnitude or even direction of change. For example, if a gene such as OTC which exhibits a complex pattern is examined at an early time following acute administration, one would conclude down-regulation by CS. On the other hand, if later time points were examined up-regulation is evident. This study also illustrates that time of exposure to drug (i.e. acute versus chronic treatment) is an important consideration to interpretation of the effects of CS on gene expression. Chronic exposure to CS in some cases results in more complex biphasic patterns of expression not evident following acute exposure, indicating probable secondary factors in control of expression. PD modeling can reveal insights into the complexities of mechanisms of gene regulation beyond simple up- or down-regulation by the drug.

In conclusion, endogenous and exogenous corticosteroids affect many physiological processes. Studying each pathway individually is more rigorous and quantitative using techniques such as Quantitative Real-Time PCR, however, it is more time consuming and laborious. Although challenges in proper analysis and understanding of microarray results have emerged since the technology evolved rapidly over the years, we were able to use this plethora of data to understand and analyze the underlying mechanism of various biological processes by using our mechanism-based PD models. The proposed models, while often premised on obvious mechanisms and supported by literature findings, also present testable hypotheses that can be examined in more extensive studies of specific genes.

## Figures and Tables

**Figure 5a:**
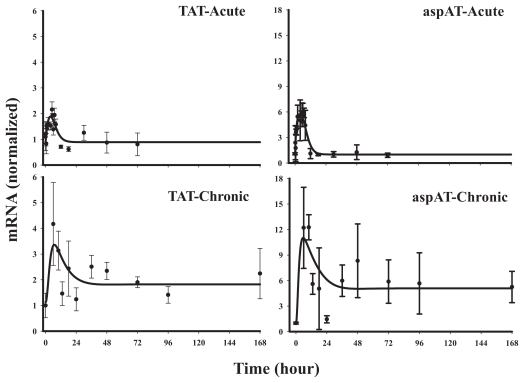
Representative fittings of *Class 1* genes Solid circles are the mean data with standard deviations. Solid lines are fittings with Model A for TAT (Eq. (11a)) and Model B for aspAT (Eq. (11b)) for each individual gene after MPL administration. The estimated parameters are listed in Table 2.

**Figure 5b:**
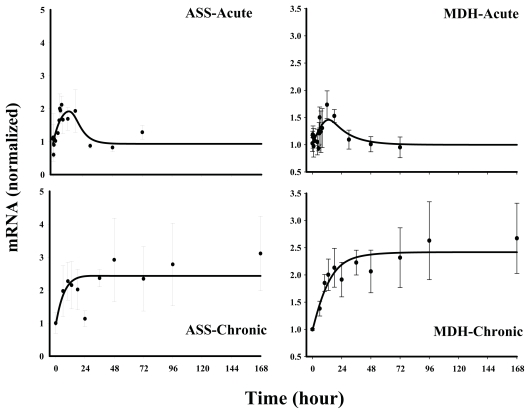
Representative fittings of *Class 1* genes Solid circles are the mean data with standard deviations. Solid lines are fittings with Model B for ASS and MDH (Eq. (11b)) for each individual gene after MPL administration. The estimated parameters are listed in Table 2.

**Figure 7:**
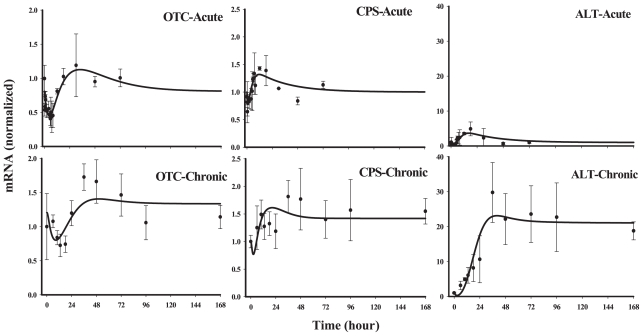
Fittings of *Class 3* genes Solid circles are the mean data with standard deviations. Solid lines are fittings with Model D (Eq. (12)–(14)) for each individual gene after MPL administration. The estimated parameters are listed in Table 4.

**Table 1:** Pharmacokinetic and receptor dynamic parameters.

Parameters	Definitions	Values
*CL (L/hr/kg)*	Clearance	5.61^a^/3.48^b^
*V_p_* (*L/kg*)	Central Volume	0.82^a^/0.73^b^
*k*_12_ (*h*^−1^)	Distribution rate constant	0.32^a^/0.98^b^
*k*_21_ (*h*^−1^)	Distribution rate constant	0.69^a^/1.78^b^
*k_syn,GRmRNA_* (*fmol/g/hr*)	GR mRNA synthesis rate constant	3.15^a^/0.45
*k_dgr,GRmRNA_*(*h*^−1^)	GR mRNA loss rate constant	0.122
*IC*_50_ (*nmol/L/mg protein*)	DR(N) required for 50% inhibition of *k_syn,GRmRNA_*	123.7
*k_on_* (*L/nmol/hr*)	Association rate constant for steroid GR binding	1.9.10^−2^
*k_T_* (*h*^−1^)	Translocation constant	58.1
*k_re_* (*h*^−1^)	Loss rate constant for DR(N)	0.402
*R_f_*	Recycling factor	0.690
*k_syn,GR_*(*nmol/L/mg protein/fmole GRmRNA/g/hr*)	GR synthesis rate constant	0.84^a^/3.63^b^
*k_dgr,GR_* (*h*^−1^)	GR loss rate constant	0.0403
*mRNA*^0^_G_*_R_* (*fmol/g*)	GR mRNA baseline	25.8^a^/3.65^b^
*R*^0^ (*fmol/mg protein*)	GR baseline	540.7^a^/328.7^b^

a Acute;

b Chronic.

**Table 2:** Estimated pharmacogenomic parameters for *Class 1* genes.

Parameters	Definitions	TAT	ASP-AT	ASS	MDH
		
		Estimate (CV%)			
*k_dgr,Enz_* (*hr*^−1^)	Loss rate of gene	0.448 (32.5)	0.81 (27.5)	0.15 (62.6)	0.066 (52.9)
*S_DR(N)_* (*fmol/mg protein*)^−1^	Linear stimulation constant	0.003 (26.6)	–	–	–
*S_max_* (*fmol/mg protein*)^−1^	Maximum stimulation	–	7.5 (34.4)	1.34 (50.9)	0.95 (75.9)
*SC*_50_ (*nM*)	Half-maximal DR(N)	–	107.4 (72.3)	4.1 (304)	14.0 (311)
*SF*	Scaling factor	2.7 (33.9)	1.6 (18.3)	1.14 (34.7)	1.6 (29.6)
*Enz_m_*_0_*_,acute_*	Baseline-Acute	0.89 (10.7)	1.0 (8.3)	0.93 (8.0)	1.0 (5.0)
*Enz_m_*_0_*_,chronic_*	Baseline-Chronic	1.05 (7.0)	1.02 (19.2)	0.97(16.1)	1.0 (5.0)

**Table 3:** Estimated pharmacogenomic parameters for *Class 2* genes.

Parameters	Definitions	ODC	ASL	CEBP
		
		Estimate (CV%)		
*k_e_* (*hr*^−1^)	Transduction rate constant	0.05 (8.7)	0.026 (35.7)	0.1 (fixed)
*k_dgr,Enz_* (*hr*^−1^)	Loss rate of gene	0.146 (15.2)	0.413 (27.4)	0.074 (21.5)
*S* (*fmol/mg protein*)^−1^	Linear stimulation constant	0.06 (16.8)	0.009 (16.7)	0.04 (22.4)
γ	Hill coefficient	13.6 (28.0)	3.8 (121)	5.2 (22.8)
*IC*_50_ (*nM*)	Half-maximal DR(N)	66.1 (2.0)	76.0 (34.3)	70.4 (9.1)
*SF*	Scaling factor	0.70 (8.4)	1.5 (13.1)	1.1 (11.9)
*Enz_m_*_0_*_,acute_*	Baseline-Acute	1 (fixed)	1 (fixed)	1 (fixed)
*Enz_m_*_0_*_,chronic_*	Baseline-Chronic	0.99 (10.4)	1 (fixed)	1 (fixed)

**Table 4:** Estimated pharmacodynamic parameters for *Class 3* genes.

Parameters	Definitions	CPS	ALT	OTC	ARG	GDH
		
		Estimate (CV%)				
*k_e_* (*h*^−1^)	Transduction rate constant	1.01 (44.3)	0.034 (15.6)	0.064 (124)	0.23 (42.1)	10 (fixed)
*k_dgr,Enz_* (*h*^−1^)	Loss rate of gene	0.039 (64.7)	0.521 (47.6)	0.04 (178)	1.75 (117)	0.25 (57.2)
*S_BS_* (*fmol/mg protein*)^−1^	Linear stimulation constant by BS	0.027 (84.8)	0.044 (27.6)	0.015 (132)	–	–
*S_max_* (*fmol/mg protein*)^−1^	Maximum stimulation	–	–	–	1.5 (38.8)	2.64 (54.2)
*SC*_50_ (*nM*)	Half-maximal DR(N)	–	–	–	11.0 (fixed)	100 (fixed)
*S_DR_*_(_*_N_*_)_ (*fmol/mg protein*)^−1^	Linear stimulation constant by DR(N)	0.023 (92.8)	0.004 (31.9)	0.012 (172)	0.005 (38)	0.008 (50.1)
*SF*	Scaling factor	3.5 (37.7)	3.2 (14.7)	1.24 (42.1)	1.9 (24.7)	2.59 (36.6)
*Enz_m_*_0_*_,acute_*	Baseline-Acute	1 (fixed)	1 (fixed)	0.81 (7.03)	1 (fixed)	1 (fixed)
*Enz_m_*_0_*_, chronic_*	Baseline-Chronic	1 (fixed)	1 (fixed)	1.16 (8.7)	1 (fixed)	1 (fixed)

**Table 5:** Estimated pharmacodynamic parameters for *Class 4* genes.

Parameters	Definitions	GR	CA	GA
		
		Estimate (CV%)		
*k_dgr,GR_* (*h*^−1^)	Loss rate of gene	0.16 (21.8)	1.39 (27.6)	0.13 (12.2)
*IC*_50_ (*fmol/mg protein*)	DR(N) for half-maximal inhibition	162.8 (36.8)	385.1 (14.9)	14.92 (56)
*SF*	Scaling factor	1.09 (40.6)	–	–
*GR_m_*_0_*_,acute_*	Baseline-Acute	1.0 (fixed)	1 (fixed)	1 (fixed)
*GR_m_*_0_*_,chronic_*	Baseline-Chronic	1.26 (8.9)	–	–
